# Spinal nerve root sleeve cysticercosis: a case report and  review of the literature

**DOI:** 10.1186/s13256-022-03733-9

**Published:** 2023-02-22

**Authors:** Benzhang Tao, Teng Li, Kaipeng Ji, Aijia Shang

**Affiliations:** 1 Department of Neurosurgery, Chinese PLA General Hospital, 28 Fuxing Road, Beijing, 100853 China; 2 Tianjin Medical University, Tianjin, China; 3 Department of Neurosurgery, Jin Cheng Da Hospital, Jincheng, Shanxi China

**Keywords:** Neurocysticercosis, Spinal cysticercosis, Subarachnoid space, Spinal nerve roots

## Abstract

**Background:**

Neurocysticercosis is a parasitic infection of the central nervous system by tapeworm larvae. Spinal cysticercosis is thought to be relatively rare, and spinal nerve root sleeve cysticercosis have not been reported previously.

**Case presentation:**

A 46-year-old Chinese Han female patient presented with low back pain and radicular pain of the right lower limb. The visual analog scale was 6. Magnetic resonance imaging showed a subarachnoid cyst at the S1 level, with a slight enhanced rim. The patient underwent surgical treatment. During surgery, we found the cyst located mainly in the subarachnoid space and partly in a sacral nerve root sleeve. Cysticercosis was also confirmed by postoperative pathological examination. Postoperative drug therapy was performed after cysticercosis was confirmed. Postoperatively, the patient was treated with oral albendazole (15 mg/kg) for 1 month. Only mild sensory impairment was left when she was discharged. After 3 years of follow-up, the visual analog scale reduced from 6 to 2, and the patient’s sensory function completely recovered. Magnetic resonance imaging showed no recurrence of cysticercosis.

**Conclusion:**

Subarachnoid cysticercosis may extend to nerve root sleeve causing back pain and radiculopathy, which may present with similar magnetic resonance imaging manifestations to Tarlov cysts. Hence, spinal subarachnoid cysticercosis should be considered as an important differential diagnosis of arachnoid cyst and sacral Tarlov cyst. Combined treatment with surgical removal and drug therapy is effective to manage spinal subarachnoid cysticercosis.

## Introduction

Neurocysticercosis is the most common parasitic infection of the central nervous system (CNS). Cysticercosis is often found intracranially; however, spinal involvement is relatively rare. Spinal cysticercosis accounts for 0.7–11.1% of all neurocysticercosis cases [1, 2]. There are three types of spinal cysticercosis: intramedullary, intradural–extramedullary (subarachnoid), and extradural. The most common type is spinal subarachnoid cysticercosis, which is frequently associated with intracranial involvement and thought to be disseminated cysticercosis [3, 4]. Primary spinal cysticercosis without intracranial involvement is sporadically reported with an extremely rare incidence, and may be caused by hematogenous dissemination.

In the present report, we aim to present a rare spinal nerve root sleeve cysticercosis that has not been reported before, and discuss the disease characteristic, diagnosis principles, and treatment based on the case report and literature review.

## Case presentation

**Fig. 1:**
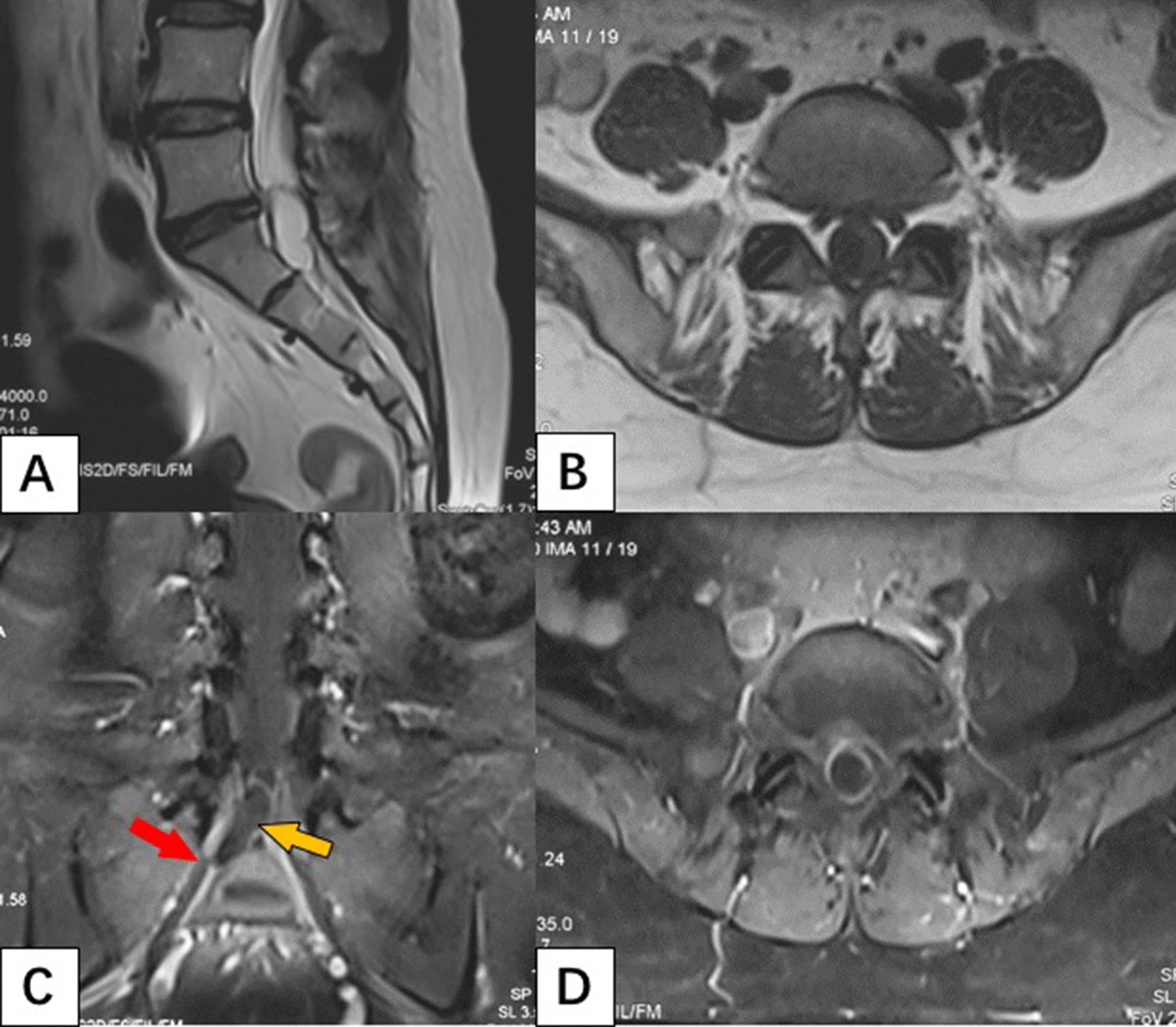
Sagittal T2-weighted MR image showing a relatively well-defined cystic intramedullary lesion on lumbosacral spine with hyperintense on T2WI (**A**). The lesion was located to the right of the spinal canal on axial T1-weighted MR with hyperintense T1WI (**B**). Coronal-enhanced T1-weighted MRI scan showed that most of the lesions (yellow arrow) were located under the dura, and some penetrated with the sleeve of L5 nerve (red arrow) (**C**). Axial-enhanced T1-weighted MR showed thin enhancing peripheral wall (**D**)

A 46-year-old Chinese Han female patient presented with low back pain and radicular pain of the right lower limb. The pain had afflicted the woman for 3 years, and gradually deteriorated. The visual analog scale (VAS) was 6 before admission to our department. Lumbar-sacral magnetic resonance imaging (MRI) demonstrated a cystic lesion in subarachnoid spaces at the S1 level, with similar signal to the cerebrospinal fluid. Part of the lesion extended into the right L5 nerve sleeve on coronal scan imaging. There was a slight enhancement of the lesion rim on a contrast-enhanced scan (Fig. 1). Brain MRI scan showed no abnormalities. The preliminary imaging diagnosis was an uncharacteristic arachnoid cyst.

**Fig. 2:**
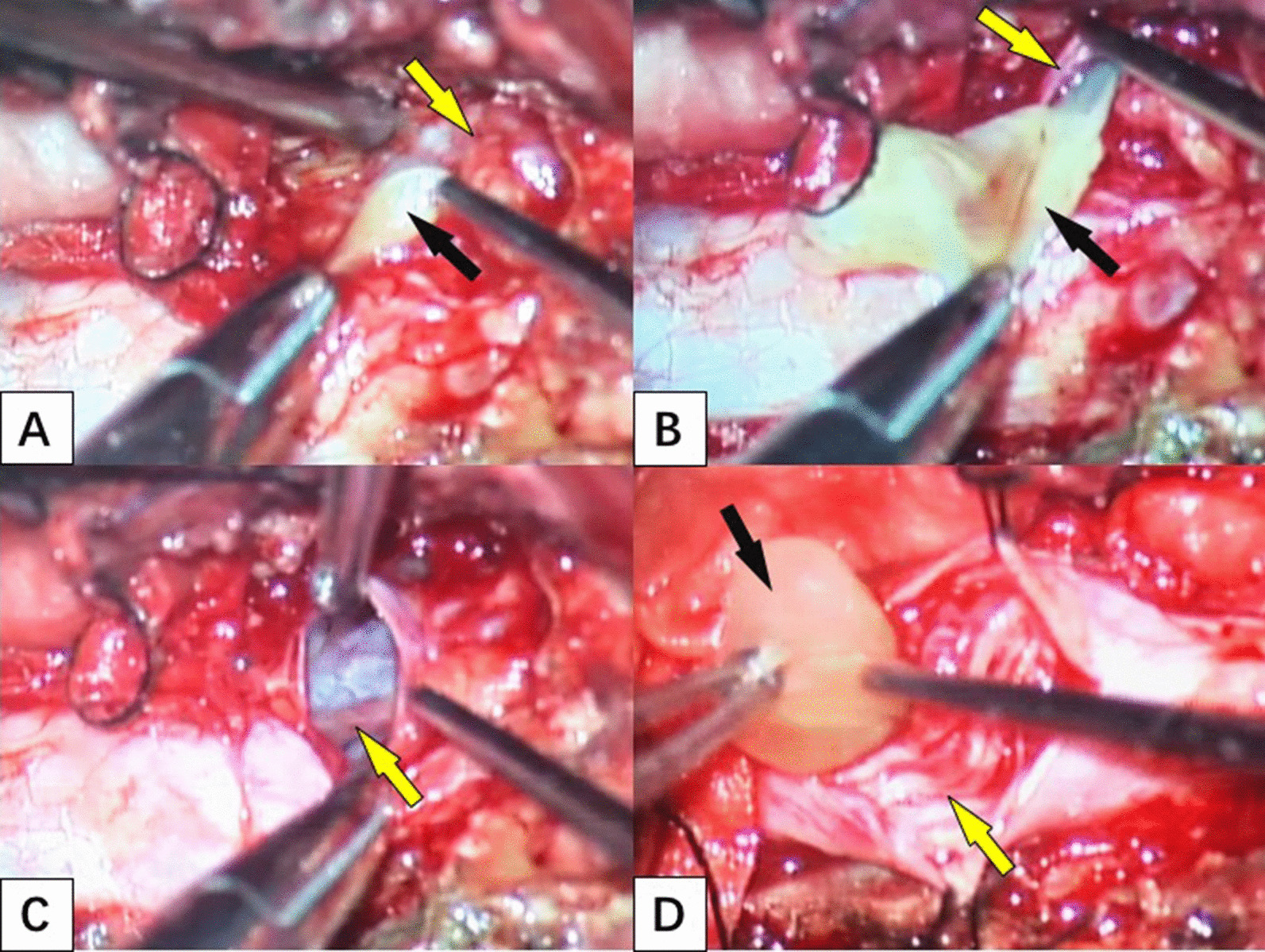
**A** incision of the nerve sleeve (yellow arrow) to expose the cystic lesion (black arrow); **B** separation of the lesion and complete removal from the nerve sleeve; **C** the nerve root (yellow arrow) is clearly displayed and intact after resection of the lesion in the nerve sleeve without cerebrospinal fluid outflow; **D** resection of the subdural lesion (black arrow) and peripheral cauda equina nerve after incision of the dura mater (yellow arrow)

Subdural cyst removal was performed. During surgery, a big, well-capsulated cyst was found after the sacral nerve sleeve was opened along its longitudinal midline. Several cysts were found after dura opening, which were yellowish and grape like. Adjacent nerves, arachnoid membrane, and dura adhered severely (Fig. 2). All the cysts were completely and integrally removed. Excision of the incrassated arachnoid membrane and release of adhesion was performed after the removal. The wound was irrigated with normal saline before closing.

**Fig. 3:**
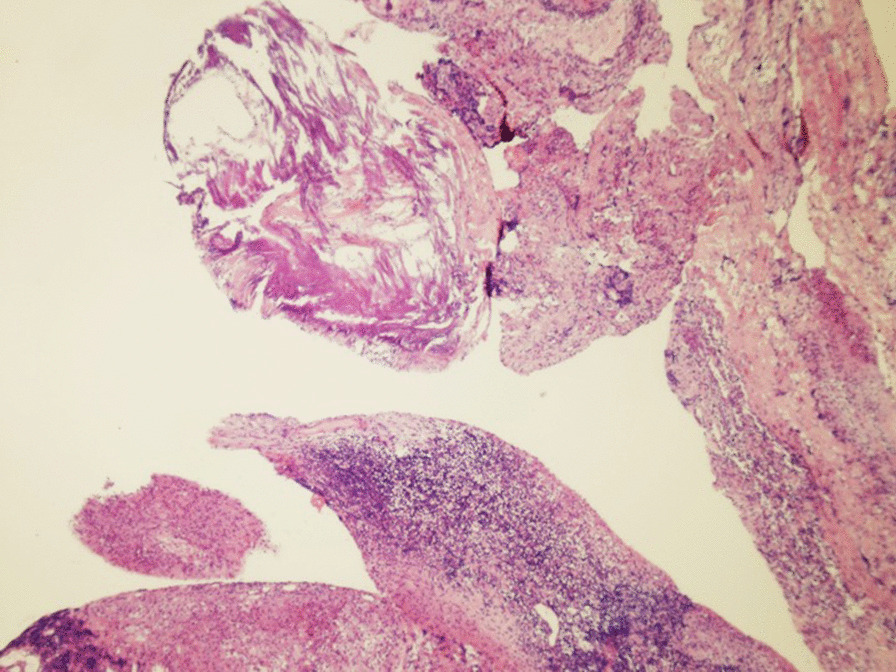
Thin cyst wall, edema, chronic inflammatory cell infiltration, and scattered calcification were found on histopathological examination, which is consistent with cysticercosis from morphology

The patient was treated with albendazole immediately after the diagnosis of cysticercosis was confirmed by pathologic examination (Fig. 3). Postoperatively, the patient was treated with oral albendazole (15 mg/kg) for 1 month. Only mild sensory impairment was left when she was discharged. After the following 3-years follow-up, VAS reduced from 6 to 2, and the patient’s sensory function completely recovered. MRI showed no recurrence of cysticercosis.

## Literature review

Cysticercosis caused by the larval form of *Taenia solium* is the most common parasitic infection in humans [1]. The CNS, eyes, skeletal muscles, and subcutaneous tissues are usually involved [1]. Humans cysticercosis are acquired by eating undercooked pork containing *T. solium* cysts or other food polluted by *T. solium* eggs [5]. When eggs are consumed by humans, oncospheres are released in the digestive tract [5]. Oncospheres can enter the bloodstream and migrate to different organs such as eyes, brain, and muscles, where they turn into the larvae form and cause the infection of cysticercosis [6].

A review of the literature showed that the clinical features of cysticercosis are often nonspecific depending on the location, number, and inflammation associated with the cysts. Inflammation usually follows larval degeneration, causing perilesional edema and mass effect, leptomeningitis, vascular compromise, and the resulting focal neurological symptoms [7]. Cervical and thoracic extramedullary cysticercosis usually induces symptoms of weakness and paresthesia by spinal cord compression. Lumbar subarachnoid cysticercosis initiate the symptoms of radicular pain of lower limbs, paresthesia, defecate abnormalities, and dysuria as a consequence of cauda equina compression. Cysticercosis may cause arachnoiditis and adhesion of nerves that induce severe pain [8, 9]. Moreover, disturbances to the absorption of cerebellar spinal fluid occasionally occurring in extensive spinal subarachnoid cysticercosis may lead to hydrocephalus.

The diagnosis of isolated intramedullary lesions can be challenging, and the differentials include a variety of cysts and neoplastic, inflammatory, demyelinating, vascular, and granulomatous lesions [8, 10–12]. Concomitant intracranial lesions can help establish a diagnosis. However, if there is no corresponding epidemiological history investigation, doctors usually ignore the existence of cysticercosis disease. Reviewing the correlations in the literature, there are two characteristics of this disease doctors need to realize. First, intraspinal cysticercosis usually presents with lesions as cysts with cerebrospinal fluid (CSF) intensity and thin enhancing peripheral wall consistent with cyst degeneration on MR imaging [13, 14]. Hence, a thin enhancing peripheral wall in T1‑weighted contrast enhancement images should be highly suspected for cysticercosis, which should be further verified during operation. Second, spinal intramedullary cysticercosis could exist in various locations, such as in the dorsal cord, conus region, and cervicodorsal region, and spinal distribution is 34% in cervical, 44.5% in thoracic, 15.5% in lumbar, and 6% in sacral regions [8, 9, 15].

Two possible explanations for the mode of entry of the parasite into the spinal cord have been reported. Most of spinal extramedullary cysticercosis occurred through cerebrospinal fluid (CSF) dissemination from intracranial CSF space lesions [16].While rare, isolated spinal intramedullary involvement by neurocysticercosis may occur, probably due to the cysticercus invading the spinal cord through reaching the subarachnoid space from cerebral ventricles, retrograding blood flow by the vertebral and intervertebral veins, or migrating transpinally [17]. Such differences need to be further explored as the detailed mechanism is still unknown.

Treatment option for neurocysticercosis depend on the American Society for Microbiology Current Consensus Guidelines: primarily surgical treatment and anticysticercal drugs have been reported as the major choice [18]. The indication of surgical treatment is that the patient has severe spinal cord compression symptoms or progressive neurological impairment. Intraspinal exploration and cyst removal are the first choice, and surgical resection and pathological detection are also the final basis for the diagnosis of suspicious cases. Although anticysticercal drugs could increase the inflammatory response, which will aggravate the clinical symptoms of patients, it is reported that anticysticercal drugs can improve the clinical curative effect after operation [19, 20].

## Discussion

Our patient was a rare case of intraspinal cysticercosis involving the nerve root sleeve, which has not been reported until now. Such an unusual cysticercus location confuses most surgeons in the distribution. Although we did not perform a CSF examination, the patient did not have a history of cysticercosis, did not come from an endemic region, and no abnormalities were found on brain MRI scan that could not support the hypothesis that cysticercus came from the CSF circulation. The cysticercus may invade the spinal cord through reaching the subarachnoid space from vertebral and intervertebral veins. The possibility of such distribution is also consistent with previous literature reports [8, 9, 15].

Furthermore, the unusual cysticercus location also confused surgeons. Based on our experience, we think that the unusual cysticercus location is a special type of intraspinal cysticercosis. We assume that cysticercosis is not originally colonized in the nerve root sleeve, but rather it grows in the subarachnoid space and slowly grows into the nerve root sleeve eventually.

## Conclusions

Spinal cysticercosis is thought to be relatively rare, and spinal nerve root sleeve cysticercosis have not been reported before. Subarachnoid cysticercosis may extend to the nerve root sleeve causing back pain and radiculopathy, which may cause similar MRI manifestations as Tarlov cysts. Hence, spinal subarachnoid cysticercosis should be considered as an important differential diagnosis of arachnoid cysts and sacral Tarlov cysts. Total removal of the cysticercosis and postoperative medical therapy can achieve complete eradication of spinal subarachnoid cysticercosis and satisfying symptom alleviation.

## Data Availability

Not applicable.

## References

[1] Sotelo J, Guerrero V, Rubio F (1985). Neurocysticercosis: a new classification based on active and inactive forms. A study of 753 cases. Arch Intern Med.

[2] Bazan R, Hamamoto Filho PT, Luvizutto GJ, Nunes HR, Odashima NS, Dos Santos AC, Elias Júnior J, Zanini MA, Fleury A, Takayanagui OM (2016). Clinical symptoms, imaging features and cyst distribution in the cerebrospinal fluid compartments in patients with extraparenchymal neurocysticercosis. PLoS Negl Trop Dis.

[3] Paterakis KN, Kapsalaki E, Hadjigeorgiou GM, Barbanis S, Fezoulidis I, Kourtopoulos H (2007). Primary spinal intradural extramedullary cysticercosis. Surg Neurol.

[4] Wiwanitkit S, Wiwanitkit V (2015). Racemose cysticercosis: a summary of 5 reported Thai cases. J Neurosci Rural Pract.

[5] Wiwanitkit V (2020). Spinal *Taenia solium* cysticercosis. Eur Spine J.

[6] Baird RA, Wiebe S, Zunt JR, Halperin JJ, Gronseth G, Roos KL (2013). Evidence-based guideline: treatment of parenchymal neurocysticercosis: report of the Guideline Development Subcommittee of the American Academy of Neurology. Neurology.

[7] Nash TE, Garcia HH (2011). Diagnosis and treatment of neurocysticercosis. Nat Rev Neurol.

[8] Qazi Z, Ojha BK, Chandra A, Singh SK, Srivastava C, Patil TB (2014). Isolated intramedullary spinal cord cysticercosis. J Neurosci Rural Pract.

[9] Qi B, Ge P, Yang H, Bi C, Li Y (2011). Spinal intramedullary cysticercosis: a case report and literature review. Int J Med Sci.

[10] Jobanputra K, Raj K, Yu F, Agarwal A (2020). Intramedullary neurocysticercosis mimicking cord tumor. J Clin Imaging Sci.

[11] do Amaral LL, Ferreira RM, da Rocha AJ, Ferreira NP (2005). Neurocysticercosis: evaluation with advanced magnetic resonance techniques and atypical forms. Top Magn Reson Imaging.

[12] Chaurasia RN, Mishra VN, Jaiswal S. Spinal cysticercosis: an unusual presentation. BMJ Case Rep. 2015. 10.1136/bcr-2014-207966. 10.1136/bcr-2014-207966 PMC4307050 25618884

[13] Parmar H, Shah J, Patwardhan V, Patankar T, Patkar D, Muzumdar D, Prasad S, Castillo M (2001). MR imaging in intramedullary cysticercosis. Neuroradiology.

[14] Del Brutto OH, Garcia HH (2013). Intramedullary cysticercosis of the spinal cord: a review of patients evaluated with MRI. J Neurol Sci.

[15] Guedes-Corrêa JF, Macedo RC, Vaitsman RP, Mattos JG, Agra JM (2006). Intramedullary spinal cysticercosis simulating a conus medullaris tumor: case report. Arq Neuropsiquiatr.

[16] Sharma R, Garg K, Agarwal D, Garg A, Sharma MC, Sharma BS, Mahapatra AK (2017). Isolated primary intradural extramedullary spinal cysticercosis. Neurol India.

[17] Zheng X, Wang F, Wang L, Li X, Li J, Huang M, Zou Y (2022). A Rare Case of Cysticercosis Involving the Whole Spinal Canal. Acta Parasitol.

[18] García HH, Evans CA, Nash TE, Takayanagui OM, White AC, Botero D, Rajshekhar V, Tsang VC, Schantz PM, Allan JC (2002). Current consensus guidelines for treatment of neurocysticercosis. Clin Microbiol Rev.

[19] Chhiber SS, Singh B, Bansal P, Pandita KK, Razdan S, Singh J (2009). Intramedullary spinal cysticercosis cured with medical therapy: case report and review of literature. Surg Neurol.

[20] Azfar SF, Kirmani S, Badar F, Ahmad I (2011). Isolated intramedullary spinal cysticercosis in a 10-year-old female showing dramatic response with albendazole. J Pediatr Neurosci.

